# Mapping and characterization of wheat stem rust resistance genes *SrTm5* and *Sr60* from *Triticum monococcum*

**DOI:** 10.1007/s00122-017-3024-z

**Published:** 2017-11-21

**Authors:** Shisheng Chen, Yan Guo, Jordan Briggs, Felix Dubach, Shiaoman Chao, Wenjun Zhang, Matthew N. Rouse, Jorge Dubcovsky

**Affiliations:** 1 Department of Plant Sciences, University of California, Davis, CA 95616 USA; 2 Department of Plant Pathology, China Agricultural University, Beijing, 100193 China; 3 Department of Plant Pathology, University of Minnesota, St. Paul, MN 55108 USA; 4 USDA-ARS Biosciences Research Laboratory, 1605 Albrecht Blvd. N., Fargo, ND 58102 USA; 5 USDA-ARS Cereal Disease Laboratory, St. Paul, MN 55108 USA; 6 Howard Hughes Medical Institute, Chevy Chase, MD 20815 USA

## Abstract

**Key message:**

The new stem rust resistance gene *Sr60* was fine-mapped to the distal region of chromosome arm 5A^m^S, and the TTKSK-effective gene *SrTm5* could be a new allele of *Sr22.*

**Abstract:**

The emergence and spread of new virulent races of the wheat stem rust pathogen (*Puccinia graminis* f. sp. *tritici*; *Pgt*), including the Ug99 race group, is a serious threat to global wheat production. In this study, we mapped and characterized two stem rust resistance genes from diploid wheat *Triticum monococcum* accession PI 306540. We mapped *SrTm5*, a previously postulated gene effective to Ug99, on chromosome arm 7A^m^L, completely linked to *Sr22*. *SrTm5* displayed a different race specificity compared to *Sr22* indicating that they are distinct. Sequencing of the *Sr22* homolog in PI 306540 revealed a novel haplotype. Characterization of the segregating populations with *Pgt* race QFCSC revealed an additional resistance gene on chromosome arm 5A^m^S that was assigned the official name *Sr60*. This gene was also effective against races QTHJC and SCCSC but not against TTKSK (a Ug99 group race). Using two large mapping populations (4046 gametes), we mapped *Sr60* within a 0.44 cM interval flanked by sequenced-based markers *GH724575* and *CJ942731*. These two markers delimit a 54.6-kb region in *Brachypodium distachyon* chromosome 4 and a 430-kb region in the Chinese Spring reference genome. Both regions include a leucine-rich repeat protein kinase (LRRK123.1) that represents a potential candidate gene. Three CC–NBS–LRR genes were found in the colinear *Brachypodium* region but not in the wheat genome. We are currently developing a Bacterial Artificial Chromosome library of PI 306540 to determine which of these candidate genes are present in the *T. monococcum* genome and to complete the cloning of *Sr60*.

**Electronic supplementary material:**

The online version of this article (doi:10.1007/s00122-017-3024-z) contains supplementary material, which is available to authorized users.

## Introduction

The current global production of common wheat (*Triticum aestivum* L., 2*n* = 2*x* = 42) exceeds 700 million tons, providing roughly one-fifth of the calories and proteins consumed by the human population (FAOSTAT 2015). As our population continues to grow, further increases in wheat production will be required to meet food demands. One way to contribute to these increases is to minimize disease yield losses caused by pathogens.

Among the wheat pathogens, the rusts have been major threats since the early times of wheat cultivation. Among the three rust diseases, stem rust caused by the fungus *Puccinia graminis* f. sp. *tritici* (*Pgt*) can be the most destructive. In the United States, severe stem rust epidemics occurred between 1919 and 1954 causing significant yield losses (Roelfs 1978). Programs for the eradication of the alternate host barberry and for the deployment of resistant varieties contributed significantly to the reduction of wheat yield losses to this disease in the USA (Singh et al. 2015).

In 1998, a new *Pgt* race was detected in Uganda that was virulent to the widely deployed stem rust resistance genes *Sr31* and *Sr38*. The corresponding isolate, designated as Ug99 (Pretorius et al. 2000), was typed as race TTKSK using the North American stem rust nomenclature system (Jin et al. 2008; Roelfs and Martens 1988). This race has spread to more than 13 countries, raising global concerns (Singh et al. 2015). During this geographic expansion, new virulences were detected in the Ug99 race group as the pathogen encountered and overcame resistance genes *Sr24* (Jin et al. 2008), *Sr36* (Jin et al. 2009), *Sr9h* (Pretorius et al. 2012; Rouse et al. 2014) and *SrTmp* (Newcomb et al. 2016; Patpour et al. 2016).

Few resistance genes effective against the Ug99 race group are present in common wheat varieties and most of the known Ug99-effective resistance genes are from the secondary and tertiary gene pools (Singh et al. 2015). The diploid wheat species *Triticum monococcum* (genome A^m^) is a tractable source of resistance genes. Chromosomes of *T. monococcum* recombine poorly with the chromosomes of hexaploid wheat in the presence of the *Pairing homologous 1* (*Ph1*) gene. However, once this gene is deleted, the homoeologous chromosomes from these two species recombine normally (Dubcovsky et al. 1995). Five loci conferring resistance to the Ug99 race complex have been postulated in this diploid species (Rouse and Jin 2011a). Among them, *Sr35* (Saintenac et al. 2013) and *Sr22* (Steuernagel et al. 2016) have been cloned, whereas *Sr21* (Chen et al. 2015) and *SrTm4* (Briggs et al. 2015) have been genetically mapped.

*Triticum monococcum* accession PI 306540, collected in Romania, is a source of multiple stem rust resistance genes. Evaluation of progeny derived from the cross between PI 306540 and stem rust-susceptible accession PI 272557 indicated the presence of three genes effective to race TTKSK, two genes effective to race QFCSC, and one gene effective to race TTTTF (Rouse and Jin 2011a). One of the three race TTKSK-effective genes in PI 306540 was predicted to be *Sr21*, which does not confer resistance to races QFCSC or TTTTF (Chen et al. 2015). A second resistance gene in PI 306540, described as *SrTm4*, conferred resistance to races TTKSK, TTTTF and QFCSC (Briggs et al. 2015). We refer to the third gene effective to race TTKSK in PI 306540 as *SrTm5*. Our initial objective in the current study was to genetically map *SrTm5*. During the mapping of *SrTm5*, we discovered that this gene does not confer resistance to race QFCSC, and that PI 306540 possesses a fourth stem rust resistance gene. This gene was mapped in a chromosome region that did not include any previously named *Sr* genes and, therefore, was assigned the new official name *Sr60*.

## Materials and methods

### Plant materials and mapping populations

The *Pgt-*resistant *T. monococcum* subsp. *monococcum* cultivated accession PI 306540 was crossed with both cultivated accession PI 272557 and wild accession G3116 (Dubcovsky et al. 1996) to generate two mapping populations. PI 272557 does not carry any known *Pgt-*resistance genes (Rouse and Jin 2011b), so a population of 108 F_2_ plants from the cross PI 306540 × PI 272557 was used to separate *SrTm5* and *Sr60* from all other genes present in PI 306540 using molecular markers. From this population, we selected two lines carrying only *SrTm5* (Pi23-15 and Pi54-3) and two lines carrying only *Sr60* (Pi57-32 and Pi57-39), both in homozygous state.

Accession G3116 (= PI 427992) is from a different subspecies (*T. monococcum* subsp. *aegilopoides*) and is highly polymorphic compared to PI 306540. This characteristic is very useful to accelerate the development of molecular markers and the generation of high-density maps. However, since both G3116 and PI 306540 are homozygous for the presence of *Sr21*, this population can not be screened with race TTKSK. Instead, we used race QFCSC, which is known to be virulent to *Sr21* (Rouse and Jin 2011a) and to *SrTm5* but not to *SrTm4* and *Sr60* (this study). To map *Sr60*, we first separated it from *SrTm4*. We used molecular markers developed by Briggs et al. (2015) to identify 18 F_2_ plants homozygous for the absence of *SrTm4* in a population of 98 F_2_ plants from the cross PI 306540 × G3116. Finally, we selected two families that showed clear phenotypic segregation and fit a 3:1 resistant:susceptible ratio in response to race QFCSC. After the initial mapping of *Sr60*, we used flanking markers to select plants heterozygous for the *Sr60* region and used these plants to develop a second segregating population of 98 plants and a third population of 1811 plants. To generate a precise genetic map of *Sr60*, we evaluated the progenies from the plants showing informative recombination events between the markers flanking *Sr60* with *Pgt* race QFCSC.

### Evaluation for stem rust resistance

All the evaluations for *Pgt* resistance were performed at the USDA-Agricultural Research Service (USDA-ARS) Cereal Disease Laboratory following procedures described before (Rouse and Jin 2011a). For gene *Sr60*, *P. graminis* f. sp. *tritici* race QFCSC (isolate 06ND76C) was used to inoculate the parents and progeny from the two mapping populations. For gene *SrTm5*, plants from the mapping population were inoculated with race TTKSK (04KEN156/04). Infection types (ITs) were visually assessed 12–14 days after inoculation using a 0–4 scale (Stakman et al. 1962). The average sporulation areas for selected assays was quantified using the image analysis software ASSESS v.2 from the American Phytopathological Society. To determine the resistance profiles of *SrTm5* and *Sr60*, lines carrying only one of these genes were inoculated with diverse *Pgt* races TTKSK (04KEN156/04), TTKST (06KEN19v3), MCCFC (59KS19), QFCSC (06ND76C), QTHJC (75ND717C), SCCSC (09ID73-2), TRTTF (06YEM34-1), TTTTF (01MN84A-1-2), and two isolates of TKTTF (13ETH18-1 and 13GER15-1).

### Development of molecular markers

For the initial mapping of *Sr60*, we screened the parental lines PI 306540 and G3116 for polymorphisms using roughly 200 simple sequence repeat (SSR) markers (GrainGenes http://wheat.pw.usda.gov) and used the polymorphic markers to genotype the selected 18 F_2_ plants lacking *SrTm4* (see “Plant materials and mapping populations”). In addition, we genotyped the same plants using the 90,000 SNP iSelect Illumina platform (Jordan et al. 2015). Once the *Sr60* region was identified, additional markers were developed from genes located in the colinear regions of the *Brachypodium dystachium* and the *T. aestivum* genomes (IWGSC CS WGA v1.0 and Zavitan WEWSeq v.1.0). Primers were designed to amplify intronic regions, and the detected SNPs were used to develop cleaved amplified polymorphic sequence (CAPS) markers and derived cleaved amplified polymorphic sequence (dCAPS) markers (Neff et al. 1998).

The population segregating for *SrTm5* was also genotyped with the 90K SNP iSelect Illumina platform. Once we found that *SrTm5* was linked to *Sr22*, we Sanger-sequenced the *Sr22* homolog in PI 306540 and compared its sequence with the different haplotypes of *Sr22* (Steuernagel et al. 2016).

### Construction of genetic linkage maps and comparisons with genomic sequences

Genetic linkage maps were constructed using MAPMAKER EXP3.0 (Lincoln et al. 1992) using the Kosambi function and a LOD threshold of 3.0. The software MAPDRAW V2.1 was used to draw the linkage maps (Liu and Meng 2003).

Sequences flanking the SNP markers were used to BLAST the *Brachypodium distachyon* genome (JGI Phytozome v11.0, https://phytozome.jgi.doe.gov/). Coordinates in the Chinese Spring wheat genome were based on the IWGSC CS WGA v1.0 assembly (Zimin et al. 2017) and those in tetraploid wheat on the NRGene assembly of the *T. turgidum* subsp. *dicoccoides* accession Zavitan (Avni et al. 2017).

## Results

### Mapping new stem rust resistance gene *Sr60* conferring resistance to race QFCSC on chromosome arm 5A^m^S

**Fig. 1:**
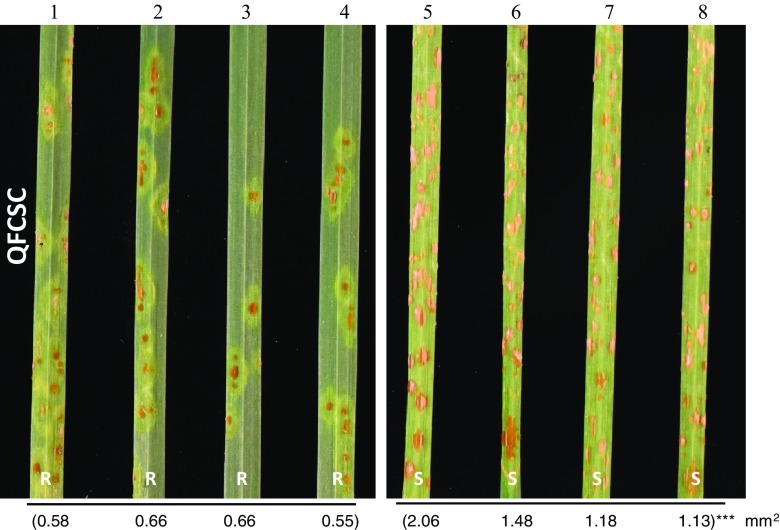
Infection types of selected PI 306540 × G3116 F_3_ families lacking *SrTm4* in response to *Puccinia graminis* f. sp. *tritici* race QFCSC (isolate 06ND76C) including families that possess *Sr60* (1–4), and families that lack *Sr60* (5–8). Numbers below leaves are average pustule size. Asterisks indicate significant differences between plants with *Sr60* and plants without this gene (***P < 0.001)

The initial mapping of *Sr60* was performed with the 18 F_2_ plants selected for the absence of *SrTm4* and their derived F_2:3_ families from the cross PI 306540 × G3116. Approximately 25 F_3_ plants from each family were phenotyped with *Pgt* race QFCSC (virulent to *Sr21* and *SrTm5* but avirulent to the new gene). We identified three families that were homozygous resistant, 12 that were segregating, and three that were homozygous susceptible (Fig. 1). These numbers were consistent with segregation of a single resistance gene (*χ* ^2^ = 2, *P* = 0.368).

**Fig. 2:**
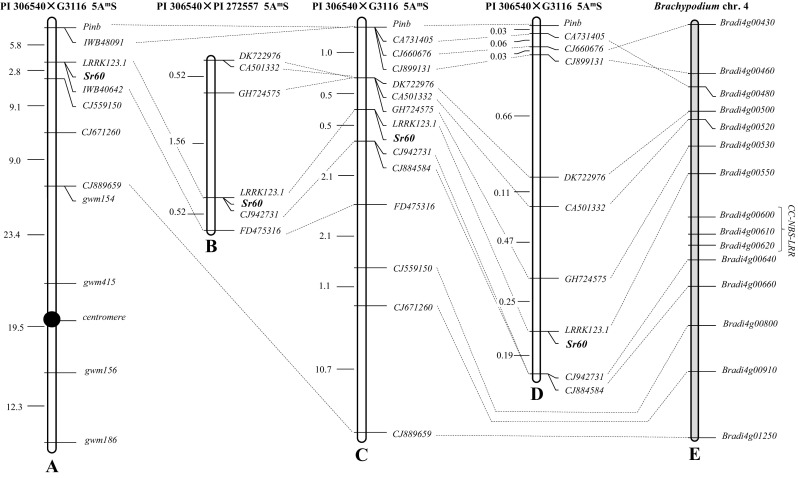
Genetic maps of *Sr60* on chromosome arm 5A^m^S. **a**, **c**, **d** Maps were derived from the cross PI 306540 × G3116 whereas **b** was derived from the cross PI 306540 × PI 272557. All phenotypes were collected from inoculations with race QFCSC. **e** Physical map of *Brachypodium* chromosome 4. **a** Initial map based on 18 F_2:3_ families lacking *SrTm4*. **b** Map based on 96 F_3:2_ plants lacking *SrTm4* and *Sr21*. **c** Map based on 98 F_3:2_ plants lacking *SrTm4*. **d** High-density map based on 1811 F_3:2_ plants lacking *SrTm4*. **e** Colinear region in the sequenced *Brachypodium* chromosome 4

Genotyping these 18 F_2_ plants with all the 90K SNP markers and polymorphic SSR markers revealed one SNP locus (*IWB40642*) completely linked to this gene (LOD score > 7) and another one (*IWB48091*) 5.8 cM distal from this gene (LOD score > 4). These two loci were previously mapped on the distal region of chromosome arm 5AS (https://triticeaetoolbox.org/wheat/). Another four SSR markers were loosely linked among each other and to *Sr60* through *gwm154*, which together with *gwm415* was located in the short arm (Fig. 2a). This last SSR marker was linked to *gwm156*, which was linked to *gwm186*, both on the long arm of chromosome 5A^m^ (Fig. 2a). These data indicated that *Sr60* was located on the distal region of the short arm of chromosome 5A^m^. Because no previously named stem rust resistance gene was mapped on chromosomes 5AS or 5A^m^S, this gene was assigned the new official name *Sr60*.

Using the sequence of SNP marker *IWB48091*, we selected *T. aestivum* contig IWGSC_chr5AS_ab_k95_contigs_longerthan_200_1551239 (https://wheat-urgi.versailles.inra.fr/Seq-Repository/BLAST). Within this contig we identified wheat gene *TraesCS5A01G003800* (marker *CJ660676*) that is orthologous to *B. distachyon* gene *Bradi4g00430*. We selected close *B. distachyon* genes (*Bradi4g00550*, *Bradi4g00800*, *Bradi4g00910* and *Bradi4g01250*) and developed markers for the corresponding wheat orthologs (*LRRK123.1*, *CJ559150*, *CJ671260*, and *CJ889659*; Table S1) that were added to the genetic map (Fig. 2a).

### Construction of a high-density genetic map of *Sr60*

The high-density map of *Sr60* was constructed in two phases. In phase one, we used a population of 98 F_3:2_ plants derived from two F_3_ families heterozygous for flanking markers *CJ660676* and *CJ889659*. Characterization of these plants with the two flanking markers and with *Pgt* race QFCSC confirmed that *Sr60* was located 1.5 cM proximal to *CJ660676* and 16.5 cM distal to *CJ889659*. To define better the position of *Sr60*, we developed and mapped nine additional molecular markers. First we developed a CAPS marker for the *Puroindoline*-*b* (*Pinb*) gene responsible for grain softness (Tranquilli et al. 1999), which is known to be located in the very distal region of the short arm of homoeologous group 5 chromosomes. This marker was completely linked to *CJ660676* (*IWB48091*), confirming its distal location. We also developed marker *FD475316* from a wheat contig selected with the sequence of *IWB40642*, and mapped it 2.6 cM proximal to *Sr60.*

We then selected seven more genes from the *Sr60* colinear region of *B. distachyon* chromosome 4 (Fig. 2e), identified the wheat orthologs and developed seven new molecular markers (*CA731405*, *CJ899131*, *DK722976*, *CA501332*, *GH724575*, *CJ942731* and *CJ884584*; Table S1) that were added to the genetic map (Fig. 2c). Based on this population, *Sr60* was completely linked to marker *LRRK123.1*, and mapped within a 1 cM interval flanked by markers *GH724575* and *CJ942731* (Fig. 2c).

In the second phase of the high-density map, we screened 1811 plants (from the same F_3_ families) segregating for flanking markers *Pinb* and *CJ884584*. We found 65 plants with recombination events within this region (1.8 cM) that were used to precisely map all the markers described above (Fig. 2d). In the final high-density map, *Sr60* remained completely linked to *LRRK123.1* and was flanked on the distal side by *GH724575* (0.25 cM) and on the proximal side by *CJ942731* (0.19 cM).

### Candidate genes for *Sr60* within the colinear regions of *B. distachyon* and wheat genomes

The high-density map of *Sr60* showed good colinearity with the genomic sequences of *B. distachyon* (Fig. 2e), and with the recent NRGene assemblies of tetraploid (*T. turgidum* subsp. *dicoccoides* var. Zavitan) and hexaploid wheat (IWGSC CS WGA v1.0). The 0.44 cM candidate region between *GH724575* and *CJ942731* in the genetic map of *T. monococcum* corresponded to a 54.6-kb region in *B. distachyon* (*Bradi4g00530*–*Bradi4g00640*) and 425- and 430-kb regions in the A genomes of tetraploid and hexaploid wheat, respectively.

There were several genes present in the *B. distachyon* genome that were not detected in the genomes of hexaploid or tetraploid wheat. Of particular interest for this project was the presence of three CC–NBS–LRR genes in the *Sr60* colinear region of *B. distachyon* (*Bradi4g00600*, *Bradi4g00610*, and *Bradi4g00620*), since this gene class is frequently associated with pathogen resistance. Although, no CC–NBS–LRR genes were detected in the colinear regions of the tetraploid and hexaploid wheat genomes (Table S2), we cannot currently rule out their presence in *T. monococcum* PI 306540.

An interesting candidate gene is *LRRK123.1* (Shumayla et al. 2016), which is located in the middle of the candidate region in the *T. monococcum* genetic map and completely linked to *Sr60*. An ortholog of this gene was found in the colinear position of *B. distachyon* (*Bradi4g00550*), which encodes a protein 91% similar to the wheat gene. The candidate wheat gene (*TraesCS5A01G005300*) is annotated in the genome of Chinese Spring (IWGSC CS WGA v1.0) as a leucine-rich repeat receptor like kinase (LRRK). We sequenced the complete *LRRK123.1* gene and found only two amino acid differences between the resistant line PI 306540 and the susceptible line PI 272557 at positions 395 and 484 (T395M and T484A; BLOSUM62 score = − 1 and 0).

### Mapping *Sr60* in the PI 306540 × PI 272557 population and selecting plants carrying only *Sr60*

We also mapped *Sr60* in a second population (PI 306540 × PI 272557) to identify plants carrying only *Sr60* and to determine its race specificity. We first characterized a population of 108 F_2_ plants from this cross with molecular markers for *Sr21* and *SrTm4* (Briggs et al. 2015; Chen et al. 2015). We identified six plants homozygous for the absence of these two genes and performed progeny tests (approximately 25 F_3_ plants per family) using *Pgt* race QFCSC. Four families segregated for resistance to race QFCSC, one was homozygous susceptible and one, designated as Pi14, was homozygous resistant.

One of the *Sr60* segregating families, designated as Pi57, was selected to generate a new mapping population of 96 F_3:2_ plants that was tested for response to QFCSC. Six of the markers mapped in the previous population (Fig. 2c, d) were polymorphic between PI 306540 and PI 272557 and were used to generate an additional genetic map for *Sr60* (Fig. 2b). In this population, *Sr60* was mapped 1.6 cM proximal to *GH724575* and 0.5 cM distal to *FD475316*, in the same interval as in the PI 306540 × G3116 population. Using these flanking markers, we confirmed that family Pi14, which was homozygous resistant to QFCSC, was also homozygous for the PI 306540 allele for the two markers flanking *Sr60.*

When we tested the progeny of Pi14 with race TTKSK, we were surprised to find a clear 3:1 segregation of the resistance response (18 resistant plants with infection type ‘;1’ and 5 susceptible plants with infection type ‘3 +’; *χ* ^2^ = 0.13, *P* = 0.72). Similar results were observed for Pi14 progeny in response to races TTKST and MCCFC. Since *SrTm5* was originally postulated based on its resistance to race TTKSK, the observed segregation in the Pi14 progeny indicated that our initial assumption that *Sr60* and *SrTm5* were the same gene was incorrect.

### Genetic map of *SrTm5* resistance using race TTKSK (Ug99)

**Fig. 3:**
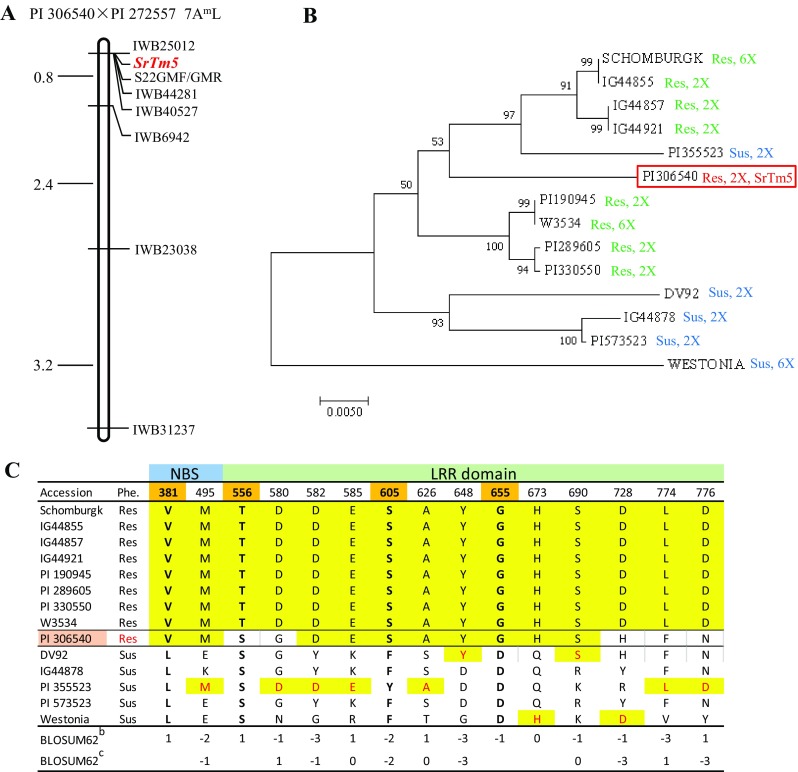
Genetic map of *SrTm5* and protein sequence relationships with previously reported *Sr22* haplotypes. **a** Genetic map of chromosome 7A^m^L. S22GMF/GMR is the *Sr22* gene-specific PCR marker and the others are SNP markers. **b** Neighbor joining phylogenetic tree of protein sequences of the NLR gene from PI 306540 linked to *Sr22* and previously characterized *Sr22* haplotypes (Steuernagel et al. 2016). *Res* resistant haplotypes, *Sus* susceptible haplotypes, *2x T. monococcum*, *6x T. aestivum.* **c** Comparison of the PI 306540 Sr22 protein haplotype with protein polymorphisms that discriminate between Sr22 resistant and susceptible haplotypes from Steuernagel et al. (2016). Positions 381, 556, 605 and 655 show a perfect discrimination between haplotypes, whereas other polymorphism included in this table have a maximum of one exception. BLOSUM62 scores are included below each amino acid position (*b* = score for the most frequent polymorphism and *c* = score for the second most frequent polymorphism)

To map *SrTm5*, we inoculated a population of 63 plants derived from line Pi14 with race TTKSK and identified 49 resistant and 14 susceptible plants. These values fit the 3:1 segregation ratio expected for a single dominant gene (*χ* ^2^ = 0.259, *P* = 0.61). Genotyping of these plants with the 90K SNP iSelect Illumina assay revealed six SNPs (*IWB25012*, *IWB44281*, *IWB40527*, *IWB6942*, *IWB23038* and *IWB31237*) significantly linked to *SrTm5* (Table 1 and Fig. 3a). Based on the linkage results, *SrTm5* was mapped to the long arm of chromosome 7A^m^ completely linked to loci *IWB25012*, *IWB44281* and *IWB40527*. Using the sequences of the linked markers, we determined that their locations in the *T. aestivum* reference genome of Chinese Spring (IWGSC CS WGA v1.0, 689,920,024–690,055,086 bp) was coincident with the location of the previously cloned gene *Sr22* (689,920,136–689,925,980 bp).

**Table 1:** SNPs linked with *SrTm5* and their previously mapped positions in hexaploid wheat

SNP ID	SNP name	Chr	Re-scaled distance cM^a^
IWB25012	Excalibur_c30730_276	7A	175.67
IWB44281	Kukri_c34147_152	7A	177.28
IWB40527	Kukri_c11141_203	7B	113.87
IWB6942	BS00022169_51	7A	178.42
IWB23038	Excalibur_c1791_819	7A	178.42
IWB31237	Excalibur_rep_c74778_252	7B	118.67

^a^Distances for the markers are from https://triticeaetoolbox.org/wheat/

To confirm the linkage between *SrTm5* and *Sr22*, we mapped the *Sr22* diagnostic marker S22GMF/GMR (Steuernagel et al. 2016) in the 63 F_2_ plants originally used to map *SrTm5* and in another 110 plants also derived from line Pi14. This marker, which amplified a 176 bp product in the resistant parent PI 306540 and no product in the susceptible parents G3116 or PI 272557, was mapped completely linked to *SrTm5* in the 173 F_2_ plants. This result suggests that *SrTm5* is either allelic to *Sr22* or is less than 1.7 cM from *Sr22* (at a 95% confidences interval) based on formulas described by Hanson (1959). We amplified the coding region of *Sr22* from PI 306540 using primers reported by Steuernagel et al. (2016). The amplified sequence (GenBank MG018615) was very similar but not identical to the six reported resistant haplotypes of *Sr22* (from 98.0 to 98.6% at the cDNA level, Table S3). A phylogenetic analysis showed that the SrTm5 predicted protein is in the middle of a cluster including Sr22 predicted proteins from different haplotypes (Fig. 3b). Only four amino acid positions discriminate perfectly the Sr22 resistant and susceptible protein haplotypes included in Fig. 3b (Fig. 3c). Among these four amino acids, PI 306540 shares three with the Sr22 resistant haplotypes and one with the susceptible haplotypes. The amino acid shared with the susceptible haplotypes (PI 306540 position 556) has a positive BLOSUM62 score of 1, which indicates a non-disruptive amino acid change. By contrast, two of the amino acids shared with the resistant haplotypes (positions 605 and 655 both in the LRR domain) have negative BLOSUM62 scores, which are indicative of changes that are more disruptive to structure and function. For the other 11 amino acid positions that show a maximum of one exception to a perfect separation between R and S haplotypes (Fig. 3c), PI 306540 has the same amino acids as the resistant haplotypes in seven of them. These results suggest that the Sr22 protein sequence present in PI 306540 is more related to the Sr22 protein in resistant haplotypes than in the susceptible haplotypes.

### Resistance profiles of *Sr60* and *SrTm5*

Using the markers linked with *Sr60* and *SrTm5*, we identified lines carrying only *Sr60* or only *SrTm5* from population PI 306540 × PI 272557. Lines Pi23-15 and Pi54-3 homozygous for *SrTm5*, and lines Pi57-32 and Pi57-39 homozygous for *Sr60* were inoculated with *Pgt* races TTKSK, TTKST (similar to TTKSK with additional virulence to *Sr24*), MCCFC, QFCSC, QTHJC, SCCSC, TKTTF (two isolates), TRTTF and TTTTF.

Plants carrying only *Sr60* displayed resistant infection types (IT = 2–22 +) to *Pgt* races QFCSC (Fig. 1), QTHJC, and SCCSC and susceptible infection types (IT = 3 +) to the other races (Table 2). We quantified the average sporulation areas using the image analysis software ASSESS v.2 and observed that the average pustule size in the plants with *Sr60* was significantly smaller than in the plants without this gene (*P* < 0.001; Fig. 1).

**Table 2:** Resistance profiles of *SrTm5* and *Sr60* to multiple *Puccinia graminis* f. sp. *tritici* races

Selected line	Resistance gene	Races								
		TTKSK	TTKST	MCCFC	QFCSC	QTHJC	SCCSC	TKTTF	TRTTF	TTTTF
Pi23-15	Only SrTm5	;1	;	1;	3 +	3 +	3 +	3 +	3 +	3 +
Pi54-3	Only SrTm5	;1	;1	;1	3 +	3 +	3 +	3 +	3 +	3 +
Pi57-32	Only Sr60	3 +	3 +	33 +	2	2	22 +	3 +	3 +	3 +
Pi57-39	Only Sr60	3 +	3 +	33 +	2	2	22 +	3 +	3 +	3 +
Pi57-57	None	3 +	3 +	3 +	3 +	3 +	3 +	3 +	3 +	3 +
Pi57-31	None	3 +	3 +	33 +	33 +	3 +	3 +	3 +	3 +	33 +

Lines were selected from the population PI 306540 × PI 272557. The two isolates of TKTTF yielded identical results

**Fig. 4:**
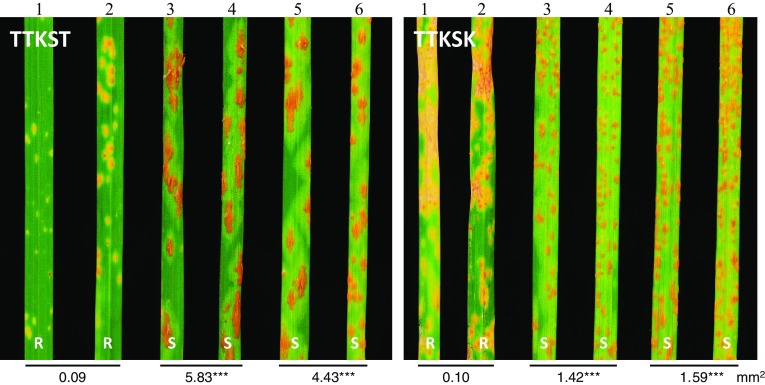
Infection types of PI 306540 × PI 272557 derivatives lines (1) Pi23-15 (*SrTm5*), (2) Pi54-3 (*SrTm5*), (3) Pi57-32 (*Sr60*), (4) Pi57-39 (*Sr60*), (5) Pi57-57 (no resistance gene) and (6) Pi57-31 (no resistance gene) in response to *Puccinia graminis* f. sp. *tritici* races TTKST and TTKSK (Ug99). *R* resistance, *S* susceptible. Numbers below leaves present the average pustule size. Asterisks indicate the existence of significant differences between plants with either *SrTm5* or *Sr60* compared to plants without either gene (***P < 0.001)

Plants carrying only *SrTm5* showed strong resistance to *Pgt* races TTKSK, TTKST and MCCFC (IT = ; to ;1) but were susceptible to the other tested races (IT = 3 +) (Fig. 4, Table 2). We also quantified the average sporulation areas in this experiment, and found the average pustule size was significantly smaller in the plants with *SrTm5* than in those without the gene (*P* < 0.001, Fig. 4). Susceptibility of plants with only *SrTm5* to races TTTTF, QFCSC, and QTHJC clearly differentiated this gene from *Sr22* that previously displayed resistance to these same races (Olson et al. 2010).

## Discussion

### Identification of new stem rust resistance gene *Sr60*

A previous study postulated that the *T. monococcum* accession PI 306540, which was collected in Romania, possessed three stem rust resistance genes (*Sr21*, *SrTm4*, and *SrTm5*) effective against *Pgt* race TTKSK (Rouse and Jin 2011a). In this study, we identified a fourth stem rust resistance gene in PI 306540 that was effective against *Pgt* race QFCSC but not against TTKSK (Table 2). This fourth gene was mapped on the distal region of chromosome arm 5A^m^S, where no previously named stem rust resistance gene has been mapped. Based on this unique chromosome location and characteristic resistant profile, this gene from PI 306540 was assigned the official designation *Sr60*.

Using our high-density genetic map, and the recently released genomes of tetraploid and hexaploid wheat (Avni et al. 2017; Zimin et al. 2017), we were able to delimit the *Sr60* candidate region to a 430-kb region in hexaploid wheat Chinese Spring and a 425-kb region in tetraploid wheat Zavitan. Within these regions we identified a leucine-rich repeat receptor like kinase *LRRK123.1*, which was completely linked to *Sr60*. Members of this gene family have been associated with disease resistance in other organisms (Chinchilla et al. 2007; Halter et al. 2014; Roux et al. 2011; Sun et al. 2004). We detected two amino acid changes in the kinase domain of LRRK123.1 between the resistant line PI 306540 and the susceptible line PI 272557, but we currently do not know if these changes affect the function of this gene. Complementation and mutant experiments will be required to demonstrate if this gene is *Sr60.*

Another class of genes frequently associated with race-specific resistance mechanisms includes the NLR genes. Many of these genes recognize effectors delivered by the pathogens or the modifications they induce in targeted host proteins and, therefore, mutations or deletions of these effectors result in susceptibility and in differences in virulence among races. Although the *Sr60* candidate regions in tetraploid and hexaploid wheat do not include any NLR genes, the presence of three NLR genes in the 54.6-kb colinear region in *B. distachyon* (*Bradi4g00600*, *Bradi4g00610*, and *Bradi4g00620*, Table S2) raises the possibility of an ancestral NLR cluster in this region that was deleted in tetraploid and hexaploid wheat. If this hypothesis is correct, we cannot rule out the possibility that some NLR genes of this cluster are still present in *T. monococcum* PI 306540. To address this possibility, we have initiated the development of a Bacterial Artificial Chromosome (BAC) library from PI 306540.

*Sr60* conferred intermediate levels of resistance to *Pgt* races QFCSC, QTHJC and SCCSC, but was susceptible to TTKSK and TTKST of the Ug99 group. This resistance profile complements well the resistance profile of *SrTm5* (Table 2), providing a potential explanation for the simultaneous presence of these two genes in PI 306540. *Sr60* was not effective against emerging virulent *Pgt* races such as the Ug99 race group, race TRTTF that was reported in Yemen and Ethiopia (Olivera et al. 2012), and isolates of race TKTTF that caused epidemics of stem rust in Ethiopia and Germany in 2013 (Olivera et al. 2015, 2017). This suggests that *Sr60* has limited potential for use in agriculture in regions where these races are present. *Sr60* may be useful in combination with other *Sr* genes if *Pgt* races emerge that are virulent on the resistance genes deployed in wheat varieties, but avirulent to *Sr60.* The identification and deployment of multiple resistance genes with an increased diversity of resistance specificities has the potential to extend the durability of wheat resistance to stem rust.

### *SrTm5* is possibly a new allele of *Sr22*

Two lines of evidence suggest that *SrTm5* is possibly a new allele of *Sr22.* First, *SrTm5* was completely linked to the diagnostic marker for *Sr22* (Fig. 3a). However, given the size of the mapping population we can only say that the two genes are less than 1.7 cM apart with a 95% confidence. Second, the sequence of the only NLR gene linked to *SrTm5* is very similar (98–99% identical in the coding regions) to *Sr22*, and the encoded protein is within a cluster including mostly *Sr22* resistant alleles (Fig. 3b). In addition, the Sr22 protein haplotype of PI 306540 carries the same amino acids as the resistant alleles at three of the four positions that discriminate the resistant and susceptible Sr22 protein haplotypes (Fig. 3c). Although it is difficult to rule out the possibility that *SrTm5* originated from a linked gene paralogous to *Sr22*, the previous results suggest that the PI 306540 *Sr22*-like gene may be the one conferring resistance to TTKSK, TTKST and MCCFC.

*SrTm5* and *Sr22* both confer resistance to *Pgt* races TTKSK (Ug99), TTKST and MCCFC, but differ in that *SrTm5* is susceptible to races QFCSC, TRTTF, and TTTTF (Table 3). We hypothesize that the 23 amino acid differences detected between *SrTm5* and the six *Sr22* resistant haplotypes (Table S3), which are all located in the LRR region, can affect the ability of the SrTm5 protein to recognize the QFCSC, TRTTF, and TTTTF effectors or the changes produced by these effectors on their target proteins.

**Table 3:** Resistance profiles of *SrTm5* and *Sr22* to multiple *Puccinia graminis* f. sp. *tritici* races

*Pgt* race	*SrTm5,* 2*x* Pi23-15/Pi54-3	*Sr22*TB, 6*x* cv. Schormburgk^a^	*Sr22*, 2*x* PI 190945^a^	No *Sr* gene, 2*x* PI 272557^a^
TTKSK	;1	2 −	1	4
TTKST	;	Not available	Not available	Not available
MCCFC	1;	2 −	;1	4
QFCSC	3 +	;2 −	;1 −	4
TRTTF	3 +	2 −;	1;	3 +
TTTTF	3 +	2	2 −	4

Infection types for *SrTm5* are from selected lines Pi23-15 and Pi54-3, whereas *Sr22* infection types are from *T. aestivum* cultivar Schormburgk and *T. monococcum* ssp. *monococcum* accession PI 190945. PI 272557 was used as susceptible check. PI 190945 carries *Sr22* and does not carry *Sr60* or *Sr21*

*2x* diploid wheat *T. monococcum*, *6x* hexaploid wheat *T. aestivum*

^a^Based on Steuernagel et al. (2016) and Rouse and Jin (2011a). Reductions in the resistance levels have been observed for other resistance genes when transferred from a lower to a higher ploidy level (McIntosh et al. 2011)

We plan to use a PI 306540 BAC library to determine if *T. monococcum* has close *Sr22* paralogs or pseudogenes in the *Sr22* region. Complementation experiments with the PI 306540 haplotype of *Sr22* (possibly *SrTm5*) will be required to demonstrate that this gene is the one that confers resistance to races TTKSK, TTKST and MCCFC but not to races QFCSC, TRTTF and TTTTF.

*SrTm5* has been postulated to be present also in *T. monococcum* accession PI 277131-2 (Rouse and Jin 2011a). Indeed, we identified a gene in PI 277131-2 that was 100% identical to the *SrTm5* candidate gene in PI 306540.

## Conclusions

In summary, we have identified a stem rust resistance locus linked to *Sr22* that has a different resistance profile than the previously characterized *Sr22* gene. Although it is likely that this gene represents a novel allele of *Sr22*, we will keep the temporary designation *SrTm5* until allelism with *Sr22* is more conclusively demonstrated. If *SrTm5* is a new allele of *Sr22*, the new haplotype will be a useful tool to understand the specificity of *Sr22* to different effectors. In addition, we identified *Sr60,* a novel race-specific stem rust resistance gene on chromosome arm 5A^m^S. With this study, we complete the genetic characterization of the different stem rust resistance genes postulated so far in diploid wheat *T. monococcum.* This information has the potential to accelerate the deployment of these genes in the polyploid wheat species and to expand our understanding of the role of different resistance genes combinations in the adaptation of diploid wheat to this devastating pathogen.

### **Author contribution statement**

ShisC and YG evaluated the populations, analyzed the data and wrote the first draft. FD contributed to the initial stages of mapping. ShiaC performed the Illumina genotyping and analyzed the data. JB and MR designed and performed the phenotyping experiments of the project. WZ contributed crosses and sequence analyses and supervised ShisC. JD initiated the project, contributed to the genetic map and statistical analyses and generated the final version of the manuscript. All authors revised the manuscript and provided suggestions.

## Electronic supplementary material

Below is the link to the electronic supplementary material.

Supplementary material 1 (PDF 157 kb)
