# Supplementary material for: Mapping and characterization of wheat stem rust resistance genes SrTm5 and Sr60 from Triticum monococcum
Source: Theor Appl Genet. 2017 Nov 21;131(3):625–35. doi: 10.1007/s00122-017-3024-z (PMC5814525; doi:10.1007/s00122-017-3024-z)
Supplement: Supplementary file 1 — Supplementary material 1 (PDF 157 kb) [file 122_2017_3024_MOESM1_ESM.pdf]

Table S1. Sources, primer sequences, restriction enzymes, annealing temperatures and expected product sizes of the markers in the *Sr60* maps.

Marker	Source gene	Maker type	Forward primer (5'-3')	Reverse primer (5'-3')	Rest. Enz.	Ann. T(℃)	Size(bp) Sus.
Markers in PI 306540 × G3116 population							
puroindoline-b (Grain softness)	AJ242715	CAPS[a]	CCTGATAAACCCAAACGGA	TAGAATCGCAGCGACACTGT	*Bam*HI	53	761
CJ660676 (*IWB48091*)	*Bradi4g00430*	CAPS	TGAGGCTTGACATCGACAGT	CAAAGCTGCATCTCCAAGGG	*Acc*I	55	680
CJ899131	*Bradi4g00460*	CAPS	ACGACCACGAGGAGGTTACT	CGATGTGCTTGTCCATTCTT	*Sal*I	53	867
CA731405	*Bradi4g00480*	CAPS	CGAGAACTTCCCCGACCGC	TCAGGAACCCAGCAACAGTAACG	*Sty*I	56	1042
DK722976	*Bradi4g00500*	CAPS	ACCGAAGACGATGGAAGAG	TGGATCAATACCCCAGAGAG	*Hha*I	53	513
CA501332	*Bradi4g00520*	CAPS	GTGGACTTCGGTGGCTTGC	CGTCAGACATAAATGGAACAGAGGA	*Ava*II	56	450
GH724575	*Bradi4g00530*	CAPS	AGAAGAAGGACACCAGGAT	AACACCGTTAGTTTTTTGAA	*BsaA*I	53	416
CF133295 (*LRRK123.1*)	*Bradi4g00550*	CAPS	TCGGCGACCTCACTCACCT	AGTTTGCTTGTCCATTTTCTCAT	*Ple*I	55	916
CJ942731	*Bradi4g00640*	dCAPS	AGAGTAAAGTCATAACTAGGCCTGCA	ACCGACGATGCTAAAAGTCTAACA	*Pst*I	57	170
CJ884584	*Bradi4g00660*	CAPS	ATGTGGCATTTACTATCGC	CCCTGTTACCCTACACTCA	*Nci*I	52	753
FD47316 (*IWB40642*)	*Bradi2g27230*	CAPS	TTCATTGCTGGCACCACTTG	ACTTTGCACGTCCCACTTTC	*Sap*I	55	1383
CJ559150	*Bradi4g00800*	CAPS	GCGGGAATGGACATAAGGAC	AGCACCCAGCAATACGGAA	*Aci*I	52	1160
CJ671260	*Bradi4g00910*	CAPS	CAAATGCGATTGTGAATGTGA	GGTAAACGAGCAGGGAGGAT	*Spe*I	52	492
CJ889659	*Bradi4g01250*	CAPS	TTATTCTGCGTATTTGGTGG	ACCGTATATCTCGTTTGCCT	*Tsp45*I	55	525
gwm154		SSR	TCACAGAGAGAGAGGGAGGG	ATGTGTACATGTTGCCTGCA		55	
gwm415		SSR	GATCTCCCATGTCCGCC	CGACAGTCGTCACTTGCCTA		55	
gwm156		SSR	CCAACCGTGCTATTAGTCATTC	CAATGCAGGCCCTCCTAAC		60	
gwm186		SSR	GCAGAGCCTGGTTCAAAAAG	CGCCTCTAGCGAGAGCTATG		60	
Markers in PI 306540 × PI 272557 population							
DK722976	*Bradi4g00500*	CAPS	ACCGAAGACGATGGAAGAG	TGGATCAATACCCCAGAGAG	*Hha*I	53	513
CA501332	*Bradi4g00520*	CAPS	GTGGACTTCGGTGGCTTGC	CGTCAGACATAAATGGAACAGAGGA	*BsiEI*	56	450
GH724575	*Bradi4g00530*	CAPS	AGAAGAAGGACACCAGGAT	AACACCGTTAGTTTTTTGAA	*BsaA*I	53	416
CF133295 (*LRRK123.1*)	*Bradi4g00550*	CAPS	TCGGCGACCTCACTCACCT	AGTTTGCTTGTCCATTTTCTCAT	*Ple*I	55	916
CJ942731	*Bradi4g00640*	dCAPS	AGAGTAAAGTCATAACTAGGCCTGCA	ACCGACGATGCTAAAAGTCTAACA	*Pst*I	57	170
FD47316 (*IWB40642*)	*Bradi2g27230*	CAPS	TTCATTGCTGGCACCACTTG	ACTTTGCACGTCCCACTTTC	*Sap*I	55	1383

[a] Cleaved amplified polymorphic sequence. [b] Derived cleaved amplified polymorphic sequence.

Table S2. Candidate genes of *Sr60* within the colinear regions in *B. distahyon* and the Chinese Spring wheat reference genome.

Brachypodium [a]	Annotation (putative function)	Wheat marker	Wheat gene[b]
Bradi4g00530	Nitrate transporter (NRT1)	*GH724575*	*TraesCS5A01G004400*
Bradi4g00540	Cytochrome P450/oxidoreductase		
Bradi4g00550	Leucine-rich repeat protein kinase	*CF133295* (*LRRK123.1*)	*TraesCS5A01G005300*
Bradi4g00560	Hemiasterlin resistant protein		
Bradi4g00565	NO		
Bradi4g00570	Domain of unknown function		
Bradi4g00580	Calcineurin-like phosphoesterase		
Bradi4g00590	Calcineurin-like phosphoesterase		
Bradi4g00600	NBS-LRR protein		
Bradi4g00610	NBS-LRR protein		
Bradi4g00620	NBS-LRR protein		
Bradi4g00630	NO		
Bradi4g00640	Nitric-oxide synthase (NADPH)	*CJ942731*	*TraesCS5A01G006100*

[a] *Brachypodium* genome: https://phytozome.jgi.doe.gov/pz/portal.html;

[b] Wheat reference genome of Chinese Spring: https://wheat-urgi.versailles.inra.fr/Seq-Repository/BLAST.

Table S3. Polymorphisms between *SrTm5* (PI 306540) and six previously reported resistant haplotypes of *Sr22*. We showed unique amino acid in *SrTm5* that are polymorphic relative to the other six resistant haplotypes of *Sr22*.

Position [a] (protein level)	*Sr22* [b] (6 R haplotypes)	*SrTm5* (PI306540)	Protein change
556	T	S	T556S
580	D	G	D580G
723	L	R	L723R
728	D	H	D728H
751	F	C	F751C
774	L	F	L774F
776	D	N	D776N
777	D/N/Y	Q	D/N/Y777Q
798	T	S	T798S
800	P/Y	S	P/Y800S
822	K/R	S	K/R822S
824	E	R	E824R
825	A/L	V	A/L825V
827	N/R	H	N/R827H
848	L	F	L848F
850	N/H	D	N/H850D
853	E	G	E853G
878	S/G	D	S/G878D
915	N/Y	F	N/Y915F
918	H	D	H918D
921	Q	R	Q921R
926	G	D	G926D
928	T	A	T928A

[a] Position is based on protein of *SrTm5* because there are three amino acids insertion or deletion in the six reported resistant haplotypes of *Sr22*;

[b] The six reported resistant haplotypes of *Sr22* could have different amino acids at the same position.
